# Recent insights into barley and *Rhynchosporium commune* interactions

**DOI:** 10.1111/mpp.12945

**Published:** 2020-06-14

**Authors:** Xuechen Zhang, Ben Ovenden, Andrew Milgate

**Affiliations:** ^1^ NSW Department of Primary Industries Wagga Wagga Agricultural Institute Wagga Wagga NSW Australia

**Keywords:** barley breeding, disease resistance, marker‐assisted selection, quantitative trait loci, *Rhynchosporium commune*, scald

## Abstract

*Rhynchosporium commune* is the causal pathogen of scald in barley (*Hordeum vulgare*), a foliar disease that can reduce yield by up to 40% in susceptible cultivars. *R. commune* is found worldwide in all temperate growing regions and is regarded as one of the most economically important barley pathogens. It is a polycyclic pathogen with the ability to rapidly evolve new virulent strains in response to resistance genes deployed in commercial cultivars. Hence, introgression and pyramiding of different loci for resistance (qualitative or quantitative) through marker‐assisted selection is an effective way to improve scald resistance in barley. This review summarizes all 148 resistance quantitative trait loci reported at the date of submission of this review and projects them onto the barley physical map, where it is clear many loci co‐locate on chromosomes 3H and 7H. We have summarized the major named resistance loci and reiterated the renaming of *Rrs15* (CI8288) to *Rrs17*. This review provides a comprehensive resource for future discovery and breeding efforts of qualitative and quantitative scald resistance loci.

## Graphical abstract

This review provides a comprehensive resource for future discovery and breeding efforts for resistance to *Rhynchosporium commune* in barley (*Hordeum vulgare*) by summarizing all 148 published resistance quantitative trait loci.

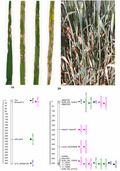

## 1 INTRODUCTION

**Figure 1:**
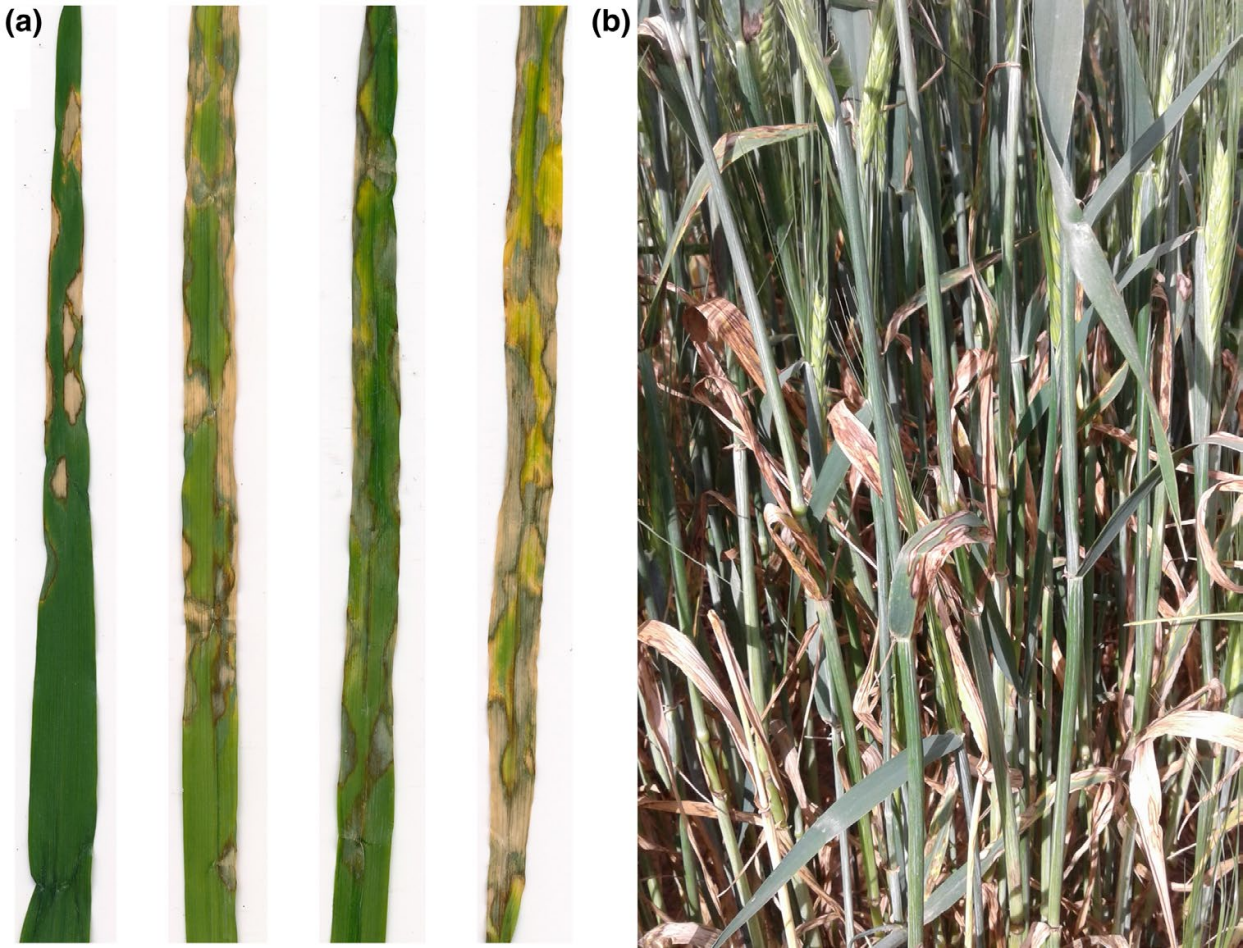
Symptoms of scald caused by *Rhynchosporium commune* on (a) individual leaves in glasshouse and (b) whole plants in the field

Scald (also known as leaf blotch) in barley (*Hordeum vulgare*) is caused by the pathogen *Rhynchosporium commune. Rhynchosporium* was first isolated from rye (*Secale cereale*) in the Netherlands (Oudemans, 1897) and thereafter named *Rhynchosporium secalis* (Oud.) J.J. Davis (Davis, 1919). Zaffarano *et al*. (2011) resolved this single species into three, with the *Rhynchosporium* infecting *Hordeum* species and *Bromus diandrus* named as a new species, *R. commune*. Scald, originally from northern Europe (Brunner *et al*., 2007), is a destructive disease of barley worldwide. It is primarily a disease of cool, humid production regions, and manifests as elongated pale blotches with a distinctive brown margin that can appear on the leaves and leaf sheath of stems (Figure 1) (Ayesu‐Offei and Carter, 1971; Zhan *et al*., 2008). Scald can cause up to 30%–40% yield loss in susceptible cultivars and also has a detrimental impact on grain quality (Paulitz and Steffenson, 2011).

Studies into the infection pathways of the pathogen have revealed there are three main routes of transmission: between seasons, across locations, and within the plant. These transmission pathways were recently reviewed by Fountaine *et al*. (2010), and Stefansson *et al*. (2012). *R. commune* survives between seasons on barley residues, and conidiophores are produced on infected crop residues, which produce spores that infect the subsequent crop. The pathogen can also be carried in the tissue of infected seeds (Brunner *et al*., 2007; Topp *et al*., 2019). Long‐distance transmission can occur by sowing infected seed or movement of stubble as hay, spreading *R. commune* to new geographical locations. As yet, the sexual form of *R. commune* is unknown, thus the possibility of long‐distance dispersal of ascospores remains as another pathway for the spread of disease. At the single leaf level, the intercellular development of the pathogen is almost exclusively restricted to the subcuticular region of host leaves (Siersleben *et al*., 2014). Transmission of the pathogen between leaf layers and plants is then driven by sporulation on the leaf surface, with spores and mycelia spreading to nearby plants via rain splash or wind.

Based on the complexity of the pathogen, control of the disease requires an integrated and multifaceted approach, including application of fungicides, manipulation of sowing date, cultural disease management, and developing disease‐resistant cultivars (Stefansson *et al*., 2012; McLean and Hollaway, 2018). Fungicides should be applied in mixtures or using alternation in modes of action to limit the rapid development of fungicide resistance (McDonald, 2015). The development of disease‐resistant cultivars is plausible as a sustainable strategy for the management of *R. commune*. However, the fungal population can change rapidly, resulting in deployed resistant cultivars and fungicides becoming ineffective after several years of commercial use (Avrova and Knogge, 2012). The objective of more stable resistance in the long term may be achieved through introgressing and pyramiding multiple different resistance loci (qualitative and quantitative) through marker‐assisted selection (MAS). In this review, the epidemiology of *R. commune* and all currently reported resistance quantitative trait loci (QTLs) are summarized. Projection of the QTLs on the barley physical map allowed the comparison of QTLs identified from different linkage maps, including multiple QTLs reported at the *Rrs1* locus on chromosome 3H and the *Rrs2* locus on chromosome 7H.

## 2 INFECTION OF BARLEY BY *R. COMMUNE*

*R. commune* fungal growth in barley occurs in four phases: germination (occurring approximately 12 hr after inoculation), then penetration (from approximately 24 hr after inoculation), leaf colonization with a slow increase in fungal biomass, followed by exponential growth with a massive gain of biomass (at around 10 days after inoculation), and a late stationary phase during which a dense stroma forms producing sporulation (Ayesu‐Offei and Clare, 1970; Zhan *et al*., 2008; Siersleben *et al*., 2014). The genus *Rhynchosporium* produces conidia from vegetative hyphae that penetrate the cuticle above host epidermal cells (Avrova and Knogge, 2012). After the formation of germ tubes from the conidia and penetration of the cuticle, thin hyphae grow mainly longitudinally along the leaf, with no growth of mycelium through stomata (Thirugnanasambandam *et al*., 2011). The thin hyphae grow rapidly in the pectin‐rich layer of the outer epidermis cell walls of the barley leaf, ultimately producing macrosymptoms and sporulation. However, *R. commune* can also show symptomless infection, where the fungus grows within the host plant without disease symptoms appearing (Walters *et al*., 2012). In one report, symptomless infection resulted in transfer of the pathogen to barley grains (Atkins *et al*., 2010).

After spore germination and cuticle penetration, susceptible genotypes experience collapse of more epidermal cells, leading to the collapse of mesophyll cells beneath extensive mycelial growth. This process gives rise to the typical final scald lesions (Lehnackers and Knogge, 1990; Thirugnanasambandam *et al*., 2011). The influence of resistance genes on infection in the case of *Rrs1* has been shown to prevent the establishment of subcuticular stroma in order to restrict *R. commune* growth (Lehnackers and Knogge, 1990). In contrast, the resistant cultivars Digger (from the UK) and Osiris (from Algeria), which both carry the *Rrs2* locus, showed larger formation of papillae and haloes in cell walls and this was presented as the key resistance mechanisms of these cultivars by Jørgensen *et al*. (1993). The physiological mechanisms underpinning genetic resistance to scald requires further elucidation with functional studies as current knowledge is limited in this area.

## 3 POPULATION GENETICS OF *R. COMMUNE*

Studies of the genetic structure of *R. commune* have revealed that the pathogen maintains high levels of genetic diversity at a micro‐scale (Linde *et al*., 2009, McDonald, 2015). For example, genetic diversity at a single geographic location (field) was found to be more than 70% of the total genetic variation in a region within Europe (Zaffarano *et al*., 2006). In a study of 265 *R. commune* isolates from Australian barley crops collected in 1996, 76% of gene diversity was distributed within the sampling site area of approximately 1 m^2^, while 19% of gene diversity was distributed among sampling sites within fields and only 5% of gene diversity was distributed among fields (McDonald *et al*., 1999). Reports of high virulence diversity in *R. commune* populations worldwide provide further evidence to suggest this pathogen is highly genetically and phenotypically diverse (McDonald *et al*., 1999; Bouajila *et al*., 2007, 2010; Stefansson *et al*., 2012, 2014).

The genetic diversity of *R. commune* populations enables it to respond rapidly to selection pressures such as the introduction of new fungicides. There are well‐documented examples of resistance developing, such as to benzimidazole fungicides, and its spread within the *R. commune* population (Locke and Phillips, 1995). In contrast, resistance to triazole fungicides evolves more slowly, resembling a quantitative decline in efficacy, which may involve different physiological mechanisms (Cooke *et al*., 2004; Zhan *et al*., 2005). Populations exposed to flusilazole, tebuconazole, and epoxiconazole previously were shown to have 10 times lower sensitivity than populations that had not previously been exposed (Robbertse *et al*., 2001; Cooke *et al*., 2004), indicating a decrease in the effectiveness of these fungicides.

Mutation and variation in the *CYP51* gene family plays a crucial role in azole fungicide resistance in a range of fungal species (Brunner *et al*., 2015). One known mechanism contributing to azole resistance in *R. commune* is the emergence of *CYP51A*, a paralog of *CYP51* that confers reduced azole sensitivity (Hawkins *et al*., 2014; Brunner *et al*., 2015; Mohd‐Assaad *et al*., 2016). *CYP51A* was present in the most azole‐resistant *R. commune* populations from New Zealand and Switzerland, indicating the influence of fungicide selection pressure on the evolution of *R. commune* populations (Mohd‐Assaad *et al*., 2016). The results of Mohd‐Assad *et al*. (2016) suggest that *CYP51A* is the most important source of fungicide resistance variation in global *R. commune* populations.

## 4 HOST–PATHOGEN INTERACTIONS

Gene‐for‐gene interactions occur with every generation of the *R. commune* life cycle between the avirulence effectors in the pathogen and corresponding resistance genes in the host (Barua *et al*., 1993). This has led to the pathogen responding rapidly after the introduction of cultivars with new resistance genes and being able to infect these cultivars with major resistance genes or a combination of genes within a few seasons (Xi *et al*., 2002). Three necrosis inducing peptides (NIP1, NIP2, and NIP3) were identified as being important during the thin hyphal forming stage in *R. commune* by Wevelsiep *et al*. (1993). Expression analysis by Kirsten *et al*. (2012) has shown that *NIP1* transcripts are present in spores, while *NIP2* and *NIP3* transcripts are synthesized after inoculation of host plants. At least two studies of diverse isolates have shown the near‐universal presence of *NIP2* and *NIP3* genes in *R. commune* populations, suggesting the importance of both of these proteins for the pathogen (Schurch *et al*., 2004; Stefansson *et al*., 2014). Mohd‐Assaad *et al*. (2019) identified two distinct *NIP1* paralogs in almost all *Rhynchosporium* species, nominated as *NIP1A* and *NIP1B*. *NIP1A* (the widely reported paralog of *NIP1*) had a more significant influence on virulence than the newly identified *NIP1B* (Mohd‐Assaad *et al*., 2019). Mohd‐Assaad *et al*. (2019) reported that *NIP1A* paralog was under significant positive selection, illustrating that *NIP1A* is the dominant effector co‐evolving with host receptors. Variation in *NIP1A* and *NIP1B* copy number was also detected across *Rhynchosporium* species (Mohd‐Assaad *et al*., 2019). Similarly, *NIP2* families of 7–10 members were found in *R. commune* isolates (Penselin *et al*., 2016). The NIP1, NIP2, and NIP3 proteins are functionally important at the early growth stages of infection, when fungal hyphae spread before the growth of dense subcuticular stroma. Production of these proteins decreases dramatically when the fungal biomass increases rapidly, suggesting the early influence of these proteins on fungal virulence (Schurch *et al*., 2004).

NIP1 functions both as an effector and an elicitor (Wevelsiep *et al*., 1993). Furthermore, Hahn *et al*. (1993) have shown that NIP1 not only is able to facilitate leaf necrosis in barley, but also induces the reactions of resistance gene *Rrs1*. The protein NIP1 is the product of the avirulence gene *AvrRrs1* (Rohe *et al*., 1995) and induces the expression of *pathogenesis‐related 10* gene in leaves of *Rrs1* barley plants (Steiner‐Lange *et al*., 2003). Schurch *et al*. (2004) have demonstrated that virulence to *Rrs1* was achieved through either a deletion or mutation of *NIP1*, but *Rrs1* does not encode for the NIP1 receptor itself. The main factor for *Rrs1*‐triggered resistance is recognition of the interaction of NIP1 with a receptor that consequently activates the plant's defence reaction (van't Slot *et al*., 2007). This makes the *NIP* genes and their presence, absence, or altered states between global populations of the pathogen a potential predictor of resistance gene effectiveness. Schurch *et al*. (2004) tested 614 different isolates from four continents, showing a *NIP1* deletion frequency of up to 45%, while *NIP2* and *NIP3* were present in almost all isolates. Isolates carrying a functional *NIP1* gene have shown significantly higher virulence than isolates where *NIP1* was nonfunctional or missing (Stefansson *et al*., 2014; Mohd‐Assaad *et al*., 2019). NIP2 and NIP3 induce necrosis in barley, but had no function as elicitors (Hahn *et al*., 1993). Strains of *R. commune* lacking *NIP1* or strains with mutated *NIP1* were shown to overcome cultivars carrying *Rrs1* (Stukenbrock and McDonald, 2009).

## 5 MAJOR GENES AND LOCI IDENTIFIED FOR SCALD RESISTANCE IN BARLEY

**Figure 2:**
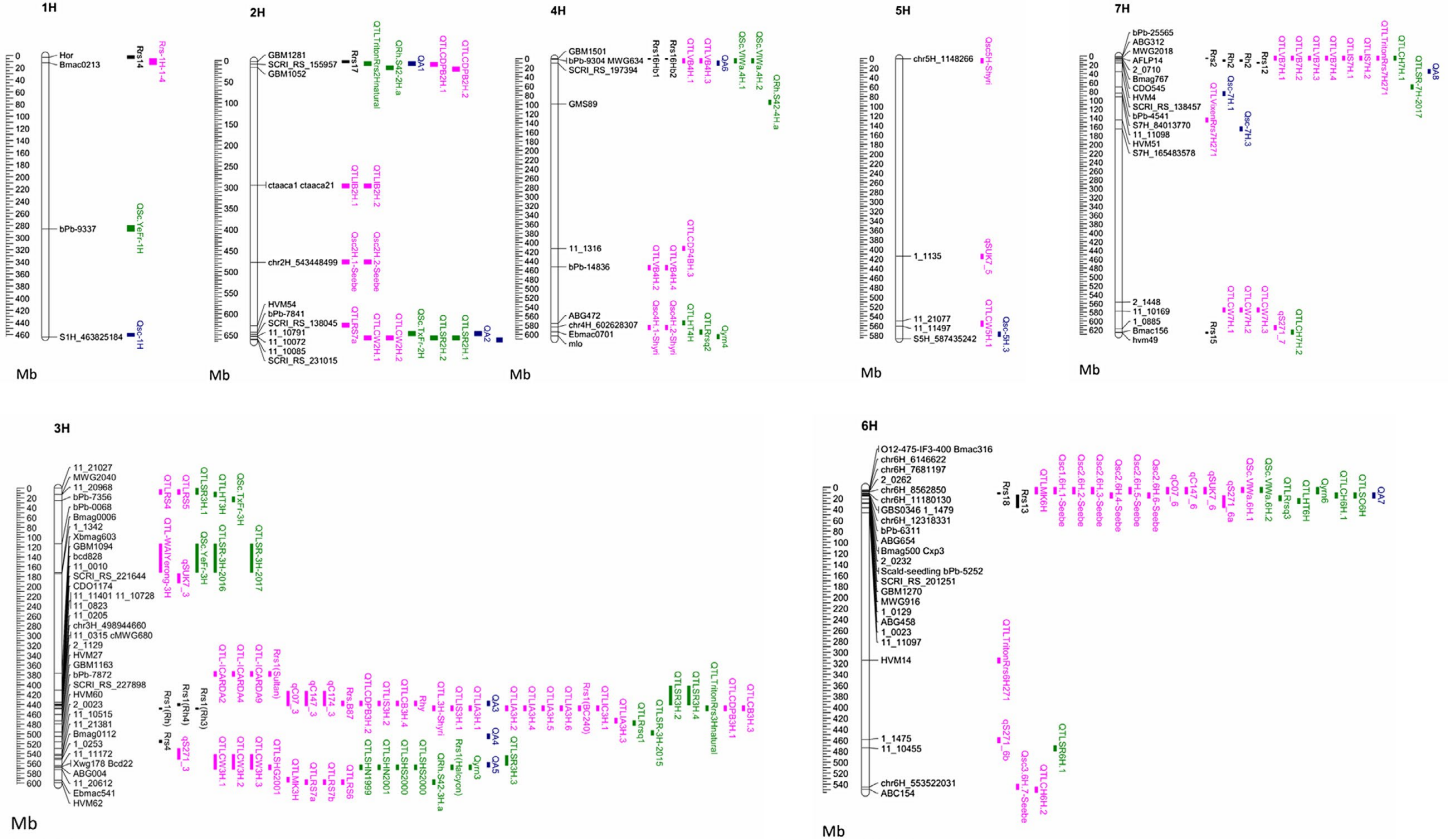
Summarized quantitative trait loci (QTLs) for scald resistance in barley on the right of chromosome (major QTL [black], QTLs identified with specific isolate inoculations in controlled environments [pink], QTLs identified under natural field conditions [green], and QTLs identified from genome‐wide association studies [blue]). Flanking markers and positions of QTLs on the barley pseudomolecules Morex v. 2.0 2019 are shown on the chromosome

The deployment of resistant cultivars and implementation of pathogen‐informed management helps reduce pesticide applications and improves long‐term crop protection. Resistance is classified into two categories: qualitative resistance genes provide high levels of resistance at all growth stages and quantitative genes provide partial levels of resistance most commonly observed at the adult plant stage (Wallwork and Grcic, 2011, Zhan *et al*., 2008). Qualitative resistance genes have been frequently identified from seedling experiments using specific isolates (Zhan *et al*., 2008). Both types of resistance are important and by pyramiding these genes together into new cultivars it is possible to create more durable disease control (Walters *et al*., 2012). Barley varieties with known sources of major resistance genes and QTLs are summarized in Table 1. The complete reference barley genome sequence enabled the comparison of QTLs identified from different genetic maps (Mascher *et al*., 2017). Overall, we have summarized 148 QTLs from 34 different studies (Table 1 and Figure 2). The sequences of markers associated with the identified QTLs for scald resistance were used to perform BLAST searches by using the IPK Barley BLAST Server (http://webblast.ipk‐gatersleben.de/barley). The barley pseudomolecules Morex v. 2.0 2019 were used for the BLASTn search (Zhang *et al*., 2019; Leng *et al*., 2020). Default settings were used to do the BLASTn search and the best hit was used to decide the physical position of the detected QTL (Table 1 and Figure 2). Most QTLs were identified using a phenotype of visually observable disease symptoms, but some studies have applied alternative methods. Looseley *et al*. (2012) detected QTLs based on the amount of *R. commune* in symptomless leaves, with results showing that the amount of *R. commune* in symptomless leaves is correlated with visual disease symptoms. Zhang *et al*. (2019) reported that a phenotype derived from the regression of disease on relative maturity is an effective trait to detect scald resistance under natural field conditions when relative maturity is correlated with scald resistance, as it reduces the possiblity of confusing maturity‐related loci with true resistance loci.

**Table 1:** Summary of scald resistance quantitative trait loci (QTLs) ordered by chromosome position giving logarithm of the odds (LOD) scores, percentage of phenotypic variation explained by the QTL in the mapping population, marker information, and the resistance source genotype

Original QTL	Chr	Physical position (Mb)	LOD	%	Closest or flanking markers	Resistant source	AGG AUS#	Agronomic characteristics of resistant source	Reference
QTLID1H*	1H	Unknown	–	–	–	Igri	401068	2 row/winter	Backes *et al*. (1995)
*Rrs14*	1H	2.8	–	–	Hor2	CPI 109829 (*Hordeum spontaneum*)	402751	Spring	Garvin *et al*. (2000)
Rrs‐1H‐1‐4	1H	12.4	46.2	86.6	Bmac0144—Bmac0213	OUH602 (*H. spontaneum*) (PI 682043)	408068		Yun *et al*. (2005)
QSc.YeFr‐1H	1H	286.2	3.7	6.4	bPb‐9337	Yerong	406299	6 row/spring	Li and Zhou (2011)
QTLTritonRrs2Hnatural	2H	10.4	13.4	38.4	GBM1281	Triton	408171	6 row/winter	Wagner *et al*. (2008)
*Rrs17* (*Rrs15* (CI8288))	2H	10.4	49.6	81.8	GBM1281	Triton	408171	6 row/winter	Wagner *et al*. (2008)
QTLCDPB2H.1*	2H	17.9	3.6	0.5	11_1175	SBCC145 (Cebada Del Pais)	411731	6 row/facultative	Hofmann *et al*. (2013)
QRh.S42‐2H.a	2H	24.2	4.2	14.6	GBM1052	ISR42‐8 (*H. spontaneum*)			von Korff *et al*. (2005)
QTLCB2H.2*	2H	28.2	2.8	1.5	11_1159	SBCC154 (Cebada)	411740	2 row/spring	Hofmann *et al*. (2013)
QTLIB2H.1*	2H	303.5	2.6	6.3	ctaaca1—cttaca6	Abyssinian (CIho 668)	400217	2 row/spring	Grønnerød *et al*. (2002)
QTLIB2H.2*	2H	303.5	2.8	5.3	ctaaca21—caaacg3	Abyssinian (CIho 668)	400217	2 row/spring	Grønnerød *et al*. (2002)
Qsc2H.2‐Seebe	2H	486.4	4.5	16.1	chr2H_543448499	Seebe	408540	2 row/spring	Zantinge *et al*. (2019)
Qsc2H.1‐Seebe	2H	486.4	4.5	23.2	chr2H_543512242	Seebe	408540	2 row/spring	Zantinge *et al*. (2019)
QTLRS7a	2H	636.1	2.5	12.2	P71m293a—HVM54	WI2291	410835	2 row/spring	Sayed *et al*. (2004)
QSc.TxFr‐2H	2H	651.7	2.9	12.8	bPb‐7841	Franklin	405994	2 row/spring	Li and Zhou (2011)
QTLCW2H.1*	2H	664.3	5.0	15.9	11_10791—11_10085	WB05‐13 (Leonie/Pearl)		2 row/winter	Looseley *et al*. (2012)
QTLCW2H.2*	2H	664.3	4.0	14.3	11_10791—11_10085	WB05‐13 (Leonie/Pearl)		2 row/winter	Looseley *et al*. (2012)
QTLSR2H.2*	2H	664.9	4.7	15.4	11_10072—11_10085	Saffron	423313	2 row/winter	Looseley *et al*. (2014)
QTLSR2H.1*	2H	664.9	5.7	16.9	11_10072—11_10085	Saffron	423313	2 row/winter	Looseley *et al*. (2014)
QTLID3H*	3H	Unknown	–	–	–	Igri	401068	2 row/winter	Backes *et al*. (1995)
QTLSR3H.1*	3H	13.5	4.6	15.2	11_21027—11_20968	Retriever	413245	2 row/winter	Looseley *et al*. (2014)
QTLRS5	3H	16.3	4.1	16.6	P294m34b—P18m184b	Tadmor	490164	2 row/spring	Sayed *et al*. (2004)
QTLRS4	3H	16.3	2.9	16.1	HVLTPPB—P71m82b	Tadmor	490164	2 row/spring	Sayed *et al*. (2004)
QTLHT3H*	3H	19.7	4.2	8.0	MWG2040	Harrington	411043	2 row/spring	Spaner *et al*. (1998)
QSc.TxFr‐3H	3H	32.3	5.2	24.6	bPb‐7356	Franklin	405994	2 row/spring	Li and Zhou (2011)
QSc.YeFr‐3H	3H	149.5	15.2	30.4	Bmag0006	Yerong	406299	6 row/spring	Li and Zhou (2011)
QTL‐WAIYerong‐3H	3H	149.5	38.7	63.5	Bmag0006	Yerong	406299	6 row/spring	Zhang *et al*. (2019)
QTLSR‐3H‐2016	3H	149.5	8.8	20.7	Bmag0006	Yerong	406299	6 row/spring	Zhang *et al*. (2019)
QTLSR‐3H‐2017	3H	149.5	4.4	9.7	Bmag0006	Yerong	406299	6 row/spring	Zhang *et al*. (2019)
qSUK7_3	3H	324.5	24.5	30.4	1_1342—2_1129	Steptoe (CIho 15,229)	411034	6 row/spring	Coulter *et al*. (2019)
QTL‐ICARDA2	3H	383.9	–	–	Xbmag603	ICARDA2			Genger *et al*. (2003b)
QTL‐ICARDA4	3H	383.9	–	–	Xbmag603	ICARDA4	406508		Genger *et al*. (2003b)
QTL‐ICARDA9	3H	383.9	–	–	Xbmag603	ICARDA9	413482		Genger *et al*. (2003b)
*Rrs1* (Sultan)	3H	383.9	–	–	Xbmag603—Xbmag225	Sultan	402019	2 row/spring	Genger *et al*. (2003b)
QTLSR3H.4*	3H	428.8	12.4	27.2	11_11401—11_10728	Retriever	413245	2 row/winter	Looseley *et al*. (2014)
QTLSR3H.2*	3H	428.8	31.3	56.2	11_11401—11_10728	Retriever	413245	2 row/winter	Looseley *et al*. (2014)
Rrs.B87	3H	442.7	–	–	bcd828	B87/14 (Selection from Pye S)	407181	2 row/spring	Williams *et al*. (2001)
QTLCDPB3H.2*	3H	446.7	73.4	84.3	11_0010	SBCC145 (Cebada Del Pais)	411731	6 row/facultative	Hofmann *et al*. (2013)
*Rrs1* (Rh4)	3H	446.9	–	–	chr3H_ 490,253,069	CIho3515 (PI 58052)	401974	6 row/winter	Looseley *et al*. (2020)
*Rrs1* (Rh4)	3H	446.9	–	–	chr3H_ 490,253,069	SBCC154 (Cebada)	411740	2 row/spring	Looseley *et al*. (2020)
*Rrs1* (Rh4)	3H	446.9	–	–	chr3H_ 490,253,069	SBCC145 (Cebada Del Pais)	411731	6 row/facultative	Looseley *et al*. (2020)
*Rrs1* (Rh4)	3H	446.9	–	–	chr3H_ 490,253,069	Retriever	413245	2 row/winter	Looseley *et al*. (2020)
*Rhy*	3H	447.3	–	–	CDO1174	E224/3 (SCRI breeding line)		Spring	Barua *et al*. (1993)
QTLIS3H.2*	3H	447.3	2.7	18.0	CDO1174—caaacc13	Steudelli (CIho 2266)	495189	2 row/spring	Bjornstad *et al*. (2004)
QTLCB3H.3*	3H	448.4	61.5	88.3	11_0823	SBCC154 (Cebada)	411740	2 row/spring	Hofmann *et al*. (2013)
QTLCB3H.4*	3H	449.9	37.8	73.1	11_0205	SBCC154 (Cebada)	411740	2 row/spring	Hofmann *et al*. (2013)
QTL.3H‐Shyri	3H	454.9	27.0	23.7	chr3H_498944660	Shyri	406671	2 row/spring	Zantinge *et al*. (2019)
QTLIA3H.5*	3H	455.3	4.9	32.6	YLM—MWG680	Abyssinian (CIho 668)	400217	2 row/spring	Grønnerød *et al*. (2002)
QTLIA3H.2*	3H	455.3	6.8	35.9	YLM—MWG680	Abyssinian (CIho 668)	400217	2 row/spring	Grønnerød *et al*. (2002)
QTLIA3H.4*	3H	455.3	13.8	72.0	YLM—MWG680	Abyssinian (CIho 668)	400217	2 row/spring	Grønnerød *et al*. (2002)
QTLIA3H.1*	3H	455.3	16.5	73.9	YLM—MWG680	Abyssinian (CIho 668)	400217	2 row/spring	Grønnerød *et al*. (2002)
QTLIA3H.6*	3H	455.3	18.3	81.5	YLM—MWG680	Abyssinian (CIho 668)	400217	2 row/spring	Grønnerød *et al*. (2002)
QTLIC3H.1*	3H	455.3	15.8	69.6	MWG680—agtc‐17	CIho 11549 (Nigri Nudum)	491320	2 row/spring	Patil *et al*. (2003)
*Rrs1* (BC240)	3H	455.3	–	–	cMWG680	CPI 109853 (*H. spontaneum*)	402768	Spring	Genger *et al*. (2003a)
QTLCDPB3H.1*	3H	455.3	141.3	96.2	11_0315	SBCC145 (Cebada Del Pais)	411731	6 row/facultative	Hofmann *et al*. (2013)
QTLIS3H.1*	3H	455.3	5.7	20.6	YLM—MWG680	Steudelli (CIho 2266)	495189	2 row/spring	Bjornstad *et al*. (2004)
*Rrs1* (Rh)	3H	455.3	–	–	cMWG680	Triton	408171	6 row/winter	Graner and Tekauz (1996)
*Rrs1* (Rh)	3H	455.3	–	–	–	Armelle	495177	2 row/spring	Looseley *et al*. (2020)
QTLTriton*Rrs3*Hnatural	3H	455.3	6.5	20.9	cMWG680	Triton	408171	6 row/winter	Wagner *et al*. (2008)
*Rrs1* (Rh4^2^)	3H	455.3	–	–	–	Modoc (CIho 7566)	495193	6 row/spring	Dyck and Schaller (1961)
*Rrs1* (Rh3)	3H	455.3	–	–	–	Turk (CIho 14400)	409324	2 row/winter	Dyck and Schaller (1961)
*Rrs1* (Rh3)	3H	455.3	–	–	–	Atlas 46 (CIho 7323)	490026	6 row/spring	Goodwin *et al*. (1990)
QTLIA3H.3*	3H	480.5	7.2	48.0	HVM27—fa1666	Abyssinian (CIho 668)	400217	2 row/spring	Grønnerød *et al*. (2002)
QTLRrsq1	3H	486.3	4.1	11.1	GBM1163	Vada (CIho 11765)	402177	2 row/spring	Shtaya *et al*. (2006)
qC07_3	3H	487.4	90.6	63.7	GBM1094—STSagtc17	CIho 3,515 (PI 58052)	401974	6 row/winter	Coulter *et al*. (2019)
qC147_3	3H	487.4	74.5	59.5	GBM1094—Bmag0112	CIho 3,515 (PI 58052)	401974	6 row/winter	Coulter *et al*. (2019)
qC174_3	3H	487.4	6.0	3.9	GBM1094—GMS0116	CIho 3,515 (PI 58052)	401974	6 row/winter	Coulter *et al*. (2019)
QTLSR‐3H‐2015	3H	503.4	5.1	14.8	bPb‐7872	Yerong	406299	6 row/spring	Zhang *et al*. (2019)
*Rrs4*	3H	523.0	15.9	69.9	HVM36b—HVM60	CIho 11549 (Nigri Nudum)	491320	2 row/spring	Patil *et al*. (2003)
qS271_3	3H	547.7	7.1	12.6	2_0023—1_0253	Morex (CIho 15773)	410940	6 row/spring	Coulter *et al*. (2019)
QTLSR3H.3*	3H	560.9	5.5	16.6	11_21381—11_11172	Saffron	423313	2 row/winter	Looseley *et al*. (2014)
QTLCW3H.3*	3H	564.0	4.0	14.3	11_10515—11_20612	Cocktail	410992	2 row/spring	Looseley *et al*. (2012)
QTLCW3H.1*	3H	564.0	20.0	38.8	11_10515—11_20612	Cocktail	410992	2 row/spring	Looseley *et al*. (2012)
QTLCW3H.2*	3H	564.0	13.0	28.1	11_10515—11_20612	Cocktail	410992	2 row/spring	Looseley *et al*. (2012)
*Rrs1* (Halcyon)	3H	573.5	28.2	59.0	Xwg178	Halcyon	411172	2 row/winter	Genger *et al*. (2003b)
QTLSHS2000*	3H	573.5	6.8	24.0	Bcd22	Halcyon	411172	2 row/winter	Read *et al*. (2003)
QTLSHN1999*	3H	573.5	8.0	52.0	Wg178	Halcyon	411172	2 row/winter	Read *et al*. (2003)
QTLSHS2000*	3H	573.5	11.0	57.0	P13/M57‐3	Halcyon	411172	2 row/winter	Read *et al*. (2003)
QTLSHN2001*	3H	573.5	13.1	48.0	P13/M57‐3	Halcyon	411172	2 row/winter	Read *et al*. (2003)
QTLSHG2001*	3H	573.5	28.3	59.0	P13/M57‐3	Halcyon	411172	2 row/winter	Read *et al*. (2003)
Qyrn3	3H	575.3	6.7	17.0	denso—ABG004	Regatta	400087	2 row/spring	Jensen *et al*. (2002)
QTLMK3H*	3H	601.4	2.1	10.0	Ebmac541	Keel	408179	2 row/spring	Cheong *et al*. (2006)
QRh.S42‐3H.a	3H	606.2	–	–	HVM62	ISR42‐8 (*H. spontaneum*)			von Korff *et al*. (2005)
QTLRS7a	3H	606.2	2.5	12.2	P71m291g—HVM62	WI2291	410835	2 row/spring	Sayed *et al*. (2004)
QTLRS7b	3H	606.2	2.5	11.9	P71m291g—HVM62	WI2291	410835	2 row/spring	Sayed *et al*. (2004)
QTLRS6	3H	606.2	2.6	15.5	P71m291g—HVM62	WI2291	410835	2 row/spring	Sayed *et al*. (2004)
QSc.VlWa.4H.1	4H	0.2	6.3	10.3	GBM1501	Vlamingh	411465	2 row/spring	Wang *et al*. (2014)
*Rrs16* Hb2	4H	1.7	–	–	MWG634	A17/1 (*Hordeum bulbosum*)			Pickering *et al*. (2006)
*Rrs16* Hb1	4H	1.7	–	–	MWG634	A17/1 (*H. bulbosum*)			Pickering *et al*. (2006)
QSc.VlWa.4H.2	4H	1.7	17.0	57.8	bPb‐9304	Vlamingh	411465	2 row/spring	Wang *et al*. (2014)
QTLVB4H.1*	4H	1.7	25.3	33.0	bPb‐9304	Vlamingh	411465	2 row/spring	Wallwork *et al*. (2014)
QTLVB4H.3*	4H	1.7	44.0	50.0	bPb‐9304	Vlamingh	411465	2 row/spring	Wallwork *et al*. (2014)
QRh.S42‐4H.a	4H	98.9	5.4	16.5	GMS89	Scarlett	407505	2 row/spring	von Korff *et al*. (2005)
QTLCDP4BH.3*	4H	413.4	10.6	1.2	11_1316	SBCC145 (Cebada Del Pais)	411731	6 row/facultative	Hofmann *et al*. (2013)
QTLVB4H.2*	4H	453.3	38.7	45.0	bPb‐14836	Vlamingh	411465	2 row/spring	Wallwork *et al*. (2014)
QTLVB4H.4*	4H	453.3	62.5	62.0	bPb‐14836	Vlamingh	411465	2 row/spring	Wallwork *et al*. (2014)
QTLHT4H*	4H	576.3	3.6	7.5	ABG472—MWG655C	TR306	411026	2 row/spring	Spaner *et al*. (1998)
Qsc4H.1‐Shyri	4H	584.0	3.0	13.1	chr4H_602628307	Shyri	406671	2 row/spring	Zantinge *et al*. (2019)
Qsc4H.2‐Shyri	4H	584.0	3.0	13.1	chr4H_602628310	Shyri	406671	2 row/spring	Zantinge *et al*. (2019)
QTLRrsq2	4H	594.4	3.9	8.3	Ebmac0701	Vada (CIho 11765)	402177	2 row/spring	Shtaya *et al*. (2006)
Qyrn4	4H	603.2	2.6	7.0	mlo	Alexis	411232	2 row/spring	Jensen *et al*. (2002)
Qsc5H‐Shyri	5H	0.9	3.0	15.3	chr5H_1148266	Shyri	406671	2 row/spring	Zantinge *et al*. (2019)
qSUK7_5	5H	414.2	7.2	6.9	1_1135—2_0265	Steptoe (CIho 15229)	411034	6 row/spring	Coulter *et al*. (2019)
QTLCW5H.1*	5H	555.7	3.5	13.6	11_21077—11_11497	Cocktail	410992	2 row/spring	Looseley *et al*. (2012)
QTLIB6H*	6H	Unknown	2.6	16.8	ctaaca29—ctgacg13	Abyssinian (CIho 668)	400217	2 row/spring	Grønnerød *et al*. (2002)
QTLID6H*	6H	Unknown	–	–	–	Igri	401068	2 row/winter	Backes *et al*. (1995)
QTLRrsq4	6H	Unknown	4.2	11.2	E35M55‐216	L94 (CIho 11797)	410943	2 row/spring	Shtaya *et al*. (2006)
QTLPostRrs6H271	6H	Unknown	3.6	19.7	V171100	Post (CIho 15695)	400062	6 row/winter	Wagner *et al*. (2008)
Qyrn6	6H	4.9	2.5	7.0	O12‐475—IF3‐400	Alexis	411232	2 row/spring	Jensen *et al*. (2002)
QTLMK6H*	6H	4.9	1.8	9.0	Bmac316	Keel	408179	2 row/spring	Cheong *et al*. (2006)
QSc.VlWa.6H.1	6H	6.0	12.0	30.0	Scald‐seedling	WABAR2147		2 row/spring	Wang *et al*. (2014)
QSc.VlWa.6H.2	6H	6.0	12.9	30.7	1_1166	WABAR2147		2 row/spring	Wang *et al*. (2014)
Qsc1.6H.1‐Seebe	6H	6.1	5.0	13.2	chr6H_6146622	Seebe	408540	2 row/spring	Zantinge *et al*. (2019)
Qsc2.6H.3‐Seebe	6H	7.7	12.0	64.6	chr6H_7681197	Seebe	408540	2 row/spring	Zantinge *et al*. (2019)
Qsc2.6H.2‐Seebe	6H	8.5	12.0	64.6	chr6H_8562850	Seebe	408540	2 row/spring	Zantinge *et al*. (2019)
qSUK7_6	6H	9.8	34.8	41.0	2_0262—1_1479	Steptoe (CIho 15229)	411034	6 row/spring	Coulter *et al*. (2019)
Qsc2.6H.5‐Seebe	6H	10.9	12.0	70.4	chr6H_11180130	Seebe	408540	2 row/spring	Zantinge *et al*. (2019)
qC07_6	6H	11.8	11.8	6.3	U35_24165—GBS0346	CIho 3515 (PI 58052)	401974	6 row/winter	Coulter *et al*. (2019)
qC147_6	6H	11.8	15.6	11.7	U35_24165—GBS0346	CIho 3515 (PI 58052)	401974	6 row/winter	Coulter *et al*. (2019)
*Rrs18* (qC174_6)	6H	11.8	97.1	68.9	U35_24165—U35_40281	CIho 3515 (PI 58052)	401974	6 row/winter	Coulter *et al*. (2019)
Qsc2.6H.6‐Seebe	6H	11.9	12.0	70.9	chr6H_12318331	Seebe	408540	2 row/spring	Zantinge *et al*. (2019)
Qsc2.6H.4‐Seebe	6H	12.0	12.0	45.8	bPb‐6311	Seebe	408540	2 row/spring	Zantinge *et al*. (2019)
QTLCH6H.1*	6H	13.6	2.2	10.0	ABG654	Harrington	411043	2 row/spring	Cheong *et al*. (2006)
QTLSO6H*	6H	14.1	15.6	51.0	Bmag500—mwg516	O’Connor	400188	2 row/spring	Cheong *et al*. (2006)
QTLRrsq3	6H	21.3	5.3	13.9	GBM1270	Vada (CIho 11765)	402177	2 row/spring	Shtaya *et al*. (2006)
*Rrs13*	6H	25.6	–	–	Cxp3—MWG916	BC Line 30 (CPI 71283)	405839	2 row/spring	Abbott *et al*. (1995)
*Rrs13*	6H	25.6	–	–	Cxp3—MWG916	Tantangara	407092	2 row/spring	Read *et al*. (1998) Genger *et al*. (2003b)
qS271_6a	6H	26.3	16.3	30.1	2_0232—1_0023	Steptoe (CIho 15229)	411034	6 row/spring	Coulter *et al*. (2019)
QTLHT6H*	6H	28.4	4.7	8.5	MWG916—WG223	Harrington	411043	2 row/spring	Spaner *et al*. (1998)
qS271_6b	6H	244.5	5.9	9.6	1_0129—1_1475	Morex (CIho 15773)	410940	6 row/spring	Coulter *et al*. (2019)
QTLSR6H.1*	6H	260.1	3.6	13.7	11_11097—11_10455	Saffron	423313	2 row/winter	Looseley *et al*. (2014)
QTLTritonRrs6H271	6H	314.6	3.2	10.4	HVM14	Triton	408171	6 row/winter	Wagner *et al*. (2008)
Qsc3.6H.7‐Seebe	6H	545.5	4.0	17.0	chr6H_553522031	Seebe	408540	2 row/spring	Zantinge *et al*. (2019)
QTLCH6H.2*	6H	549.2	2.6	10.0	ABC154	Chebec	406877	2 row/spring	Cheong *et al*. (2006)
QTLID7H*	7H	Unknown	–	–	–	Igri	401068	2 row/winter	Backes *et al*. (1995)
QTLIB7H*	7H	Unknown	3.7	28.8	cacacg15—ctaaca36	Ingrid	411256	2 row/spring	Grønnerød *et al*. (2002)
QTLVB7H.3*	7H	1.6	3.3	5.0	bPb‐25565	Buloke	411103	2 row/spring	Wallwork *et al*. (2014)
QTLVB7H.2*	7H	1.6	17.7	24.0	bPb‐25565	Buloke	411103	2 row/spring	Wallwork *et al*. (2014)
QTLCH7H.1*	7H	2.0	3.4	11.0	ABG312	Harrington	411043	2 row/spring	Cheong *et al*. (2006)
QTLIS7H.2*	7H	4.4	2.9	19.0	MWG2018—Bmag0206	Steudelli (CIho 2266)	495189	2 row/spring	Bjornstad *et al*. (2004)
QTLIS7H.1*	7H	4.4	5.1	20.0	MWG2018—Bmag0206	Steudelli (CIho 2266)	495189	2 row/spring	Bjornstad *et al*. (2004)
*Rrs2*	7H	5.2	10.0	50.0	AFLP14—P1D23R	Atlas (CIho 4118)	495017	6 row/spring	Hanemann *et al*. (2009)
*Rrs2*	7H	5.2	–	–	AFLP14—P1D23R	Atlas 46 (CIho 7323)	490026	6 row/spring	Goodwin *et al*. (1990)
*Rrs2*	7H	5.2	–	–	AFLP14—P1D23R	Digger	495143	2 row/spring	Hanemann *et al*. (2009)
*Rrs2*	7H	5.2	–	–	AFLP14—P1D23R	Osiris (CIho 1622)	495182	6 row/spring	Hanemann *et al*. (2009)
QTLVB7H.4*	7H	5.4	5.6	8.0	2_0710	Buloke	411103	2 row/spring	Wallwork *et al*. (2014)
QTLVB7H.1*	7H	5.4	11.2	16.0	2_0710	Buloke	411103	2 row/spring	Wallwork *et al*. (2014)
QTLTritonRrs7H271	7H	7.7	8.3	24.8	Bmag767	Triton	408171	6 row/winter	Wagner *et al*. (2008)
*Rh2*	7H	10.8	–	–	CDO545	Atlas (CIho 4118)	495017	6 row/spring	Schweizer *et al*. (1995)
*Rh2*	7H	10.8	–	–	CDO545	Atlas (CIho 4118)	495017	6 row/spring	Schweizer *et al*. (1995)
*Rrs12*	7H	16.0	–	–	Acp2 Bmag7	AB200	422096	2 row/spring	Abbott *et al*. (1992) Genger *et al*. (2003b)
QTLSR‐7H‐2017	7H	69.9	5.8	12.9	bPb‐4541	Franklin	405994	2 row/spring	Zhang *et al*. (2019)
QTLVixenRrs7H271	7H	144.8	4.0	21.6	HVM51	Vixen	400025	2 row/winter	Wagner *et al*. (2008)
QTLCW7H.2*	7H	334.9	8.0	20.5	11_11098—11_10169	Cocktail	410992	2 row/spring	Looseley *et al*. (2012)
QTLCW7H.1*	7H	334.9	10.0	23.5	11_11098—11_10169	Cocktail	410992	2 row/spring	Looseley *et al*. (2012)
QTLCW7H.3*	7H	334.9	8.0	20.5	11_11098—11_10169	Cocktail	4103992	2 row/spring	Looseley *et al*. (2012)
qS271_7	7H	587.6	4.3	7.4	2_1448—1_0885	Steptoe (CIho 15229)	411034	6 row/spring	Coulter *et al*. (2019)
QTLCH7H.2	7H	625.6	3.4	11.0	Bmac156	Harrington	411043	2 row/spring	Cheong *et al*. (2006)
*Rrs15*	7H	626.3	–	–	hvm49	BC line 35 (CPI 77132)	405844	2 row/spring	Genger *et al*. (2005)

Note

Where the resistant source genotype is held by the Australian Grains Genebank, the relevant accession number (AUS number) is provided. Agronomic information has been sourced from the Australian Grains Genebank and references where available. More information on genotypes with CIho accession numbers can be obtained from the United States Department of Agriculture Germplasm Resources Information Network (GRIN) online database (https://www.ars‐grin.gov/). Some barley breeding lines with major known scald resistance loci but unknown QTL positions are also included. Primer sequences for the markers listed can be obtained from the Grain Genes online database (https://wheat.pw.usda.gov/GG3/) or from the original reference.

* These QTLs were not specifically named in the original reference. The unique QTL names used here are derived from the names of the parent cultivars of the mapping populations. For all other QTLs, names from the original reference are used.

Genes and QTLs to *R. commune* resistance have been identified across the barley genome. However, some loci are linked with resistance repeatedly, which could suggest limited genetic diversity for resistance in modern barley germplasm. Over the past 20 years, QTL analysis has been used to reveal knowledge about the genetic architecture of *R. commune* resistance in barley germplasm and discover targets for marker‐assisted resistance breeding. The importance (and hence frequent selection) of the *Rrs1* and *Rrs2* loci for resistance has been illustrated with a range of biparental populations across many environments, including under natural field conditions and specific isolate inoculations in controlled environments (Table 1 and Figure 2). The number of major loci reported for *R. commune* resistance is limited to *Rrs1*, *Rrs2*, *Rrs4*, *Rrs12*, *Rrs13*, *Rrs14*, *Rrs15*, *Rrs16*, *Rrs17* (*Rrs15* (CI8288)), and *Rrs18* (Table 1 and Figure 2). Reasons for the relative lack of identified genes may be the close relationships among germplasm and the multiple reselections of the same allele combinations from different cultivars (Williams, 2003). The remaining 129 QTLs reported at the time of submission of this review are found across all seven barley chromosomes. We have included all reported QTLs in Table 1 with position and marker information where available and review the major QTLs below.

There is a lack of clarity in the literature around the nomenclature for the two loci both referred to as *Rrs15*. The locus on 7H reported by Genger *et al*. (2005) as *Rrs15* is referred to in an earlier review by the same authors (Genger *et al*., 2003b), but not described by name. Subsequently Schweizer *et al*. (2004) reported and named a locus on 2H as *Rrs15* (CI8288), and this name for this locus was retained by Wagner *et al*. (2008). This locus on 2H termed *Rrs15* (CI8288) was renamed by Zhan *et al*. (2008) as *Rrs17* to distinguish it from the locus on 7H, which remains named *Rrs15*. This was acknowledged by Coulter *et al*. (2019), who designated a new locus on 6H as *Rrs18*. We reiterate the name change suggested by Zhan *et al*. (2008) in this review.

On chromosome 1H, the major QTL *Rrs14* has been mapped at 2.8 Mb and could be introduced into barley cultivars in combination with other QTLs on different chromosomes (Garvin *et al*., 2000). Because this QTL is not linked to other resistance QTLs, the likelihood of recovering the relevant recombinant genotypes from an intercross population is possibly increased (Garvin *et al*., 2000). Another QTL from *Hordeum spontaneum* (Rrs‐1H‐1‐4) was identified close to *Rrs14* (Yun *et al*., 2005).

Chromosome 2H has a major QTL *Rrs17* (*Rrs15* (CI8288)) sourced from the cultivar Triton, which was located at 10.4 Mb on the barley physical map (Wagner *et al*., 2008). This QTL was identified under both natural field conditions and glasshouse conditions with specific isolate inoculation, suggesting this region could be further explored as a source of resistance.

The major feature on chromosome 3H is the *Rrs1* locus, encompassing the previously mapped loci *Rh*, *Rh1*, *Rh3*, *Rh4*, and *Rh7* (Bjornstad *et al*., 2002). Many alleles are reported for *Rrs1* (Dyck and Schaller, 1961; Baker and Larter, 1963; Habgood and Hayes, 1971; Hansen and Magnus, 1973; Bockelman *et al*., 1977; Bjornstad *et al*., 2002; Read *et al*., 2003; Genger *et al*., 2005; Hofmann *et al*., 2013). *Rrs1* (Rh4 type) has been identified in cultivars La Mesita, Trebi, Osiris, CIho3515, and Spanish landraces SBCC145 and SBCC154. A similar allele of *Rrs1* termed Rh4^2^ has been identified in the cultivar Modoc (Dyck and Schaller, 1961). In Turk and Atlas46, another resistance allele *Rrs1* (Rh3 type) was identified closely linked to the *Rrs1* (Rh4^2^ type) from Modoc (Dyck and Schaller, 1961). Graner and Tekauz (1996) showed that the dominant *Rh* locus and *Rrs1Rh* for scald resistance co‐segregated with RFLP marker cMWG680 on chromosome 3H, located at 455.3 Mb on the barley physical map. *Rrs1* (Rh4 type) (QTLCB3H.3) was fine mapped within 9 Mb in populations derived from the Spanish barley landraces SBCC145 and SBCC154 at 448.4 Mb (Hofmann *et al*., 2013). The newly identified diagnostic marker for *Rrs1* (Rh4 type) chr3H_490253069 was mapped at 446.9 Mb on chromosome 3H by using pseudomolecules Morex v. 2.0 2019 (Looseley *et al*., 2020). This marker chr3H_490253069 can distinguish *Rrs1* (Rh4 type) from other genes or alleles (for resistance and susceptibility) at the *Rrs1* locus (Looseley *et al*., 2020). The resistant *Rrs1* (Rh4 type) locus was present in cultivars SBCC145, SBCC154, CIho3515, and Retriever. Other cultivars that do not contain the *Rrs1* (Rh4) locus based on marker results in Looseley *et al*. (2020), but are reported to have another allele of *Rrs1* include Armelle (Rh type), Atlas 46 (Rh3 type), Abyssinian, CIho11549, Steudelli, and Triton (Rh type) (Table 1) (Looseley *et al*., 2020). There are 27 major or minor QTLs for scald resistance summarized at the *Rrs1* locus (spreading from 429 to 503 Mb) from both natural field experiments and isolate inoculation experiments, indicating the importance of *Rrs1* (Spaner *et al*., 1998; Williams *et al*., 2001; Grønnerød *et al*., 2002; Jensen *et al*., 2002; Sayed *et al*., 2004; Shtaya *et al*., 2006; Li and Zhou, 2011). Only five of the 27 QTLs were identified under natural field conditions. The remaining 22 were all major QTLs based on seedling resistance screens with specific isolates. *Rrs1* has been mapped as both a qualitative gene (Li and Zhou, 2011; Wagner *et al*., 2008; Looseley *et al*., 2014) and as a quantitative gene (von Korff *et al*., 2005; Shtaya *et al*., 2006) for adult plant resistance under natural field conditions. Another major QTL *Rrs4* was mapped in proximity to *Rrs1* at 523.0 Mb on chromosome 3H (Patil *et al*., 2003). With many resistance QTLs identified in the same region as the *Rrs1* locus on chromosome 3H, it remains unknown if these QTLs are alleles of the same gene, or if they are part of a closely linked gene cluster (Patil *et al*., 2002). Recently, Looseley *et al*. (2020) developed molecular markers for *Rrs1* (Rh4 type) using a collection of Syrian and Jordanian landrace lines. This study suggests that resistance at the *Rrs1* (Rh4 type) locus is controlled by a presence/absence variant that is absent in the current Morex genome sequence. Looseley *et al*. (2020) also postulated that there are relatively few independent introgressions of the *Rrs1* (Rh4 type) locus in UK barley genotypes and provided a list of 25 cultivars that contain this locus. The *Rrs16* locus is positioned on chromosome 4H at 1.7 Mb. This major QTL was introgressed into cultivated barley from interspecific crosses with *Hordeum bulbosum* (Pickering *et al*., 2006). There have been a further six QTLs located close to *Rrs16* reported from two different studies using both natural field inoculation and individual isolate screening (Wallwork *et al*., 2014; Wang *et al*., 2014). In both studies, the resistant allele for the QTLs was derived from the cultivar Vlamingh. In each study the reported QTLs have major effects explaining more than 10% phenotypic variance, with a reported logarithm of the odds (LOD) value greater than 3 (Table 1).

No major scald resistance genes have been reported on chromosome 5H. However, minor effect QTLs for scald resistance have been mapped to this chromosome (Looseley *et al*., 2012; Coulter *et al*., 2019; Zantinge *et al*., 2019).

Chromosome 6H contains the major QTL *Rrs13*, originally mapped in an interspecific cross with *H. spontaneum* (Abbott *et al*., 1995; Genger *et al*., 2003a). QTLs for scald resistance at adult plant stage were also identified at the *Rrs13* locus (Spaner *et al*., 1998; Shtaya *et al*., 2006). The Australian cultivar Tantangera was bred using the *Rrs13* source AB6 described in Abbott *et al*. (1995) by Read *et al*. (1998) and is known to contain the *H. spontaneum Rrs13* locus. A novel resistance locus *Rrs18* was mapped at 11.8 Mb on chromosome 6H (Coulter *et al*., 2019). The distance between *Rrs13* and *Rrs18* was approximately 10 Mb after projecting *Rrs13* and *Rrs18* onto the barley physical map (Coulter *et al*., 2019). *Rrs18* was further fine‐mapped to an interval of 660 kb (10.92–11.58 Mb). The position of *Rrs18* was based on the August 2015 barley pseudomolecule contigs (Coulter *et al*., 2019). By using pseudomolecules Morex v. 2.0 2019, *Rrs18* was mapped at 11.8 Mb. A putative kinase protein HORVU6Hr1G005260 containing a potential extracellular domain with a signal peptide, a transmembrane domain, and a serine/threonine kinase domain was the identified candidate gene at *Rrs18* (Coulter *et al*., 2019). Recently, a new major QTL Qsc2.6H explaining 70.9% phenotypic variance was discovered in the same region as *Rrs18* (10.9–12.0 Mb) by Zantinge *et al*. (2019).

A number of resistant QTLs have been identified on chromosome 7H. The *Rrs2* locus from Atlas was fine‐mapped to an interval of 0.08 cM positioned at 5.2 Mb on the barley physical map (Schweizer *et al*., 1995; Hanemann *et al*., 2009). The *Rrs2* resistance locus is effective against most *R. commune* isolates and is important for barley breeding programmes worldwide (Hanemann *et al*., 2009). The *Rrs12* locus introgressed from *H. spontaneum* is positioned at 16.0 Mb on chromosome 7H (Abbott *et al*., 1992). There are another nine different QTLs overlapping between *Rrs2* and *Rrs12* that have also been reported (Bjornstad *et al*., 2004; Cheong *et al*., 2006; Wagner *et al*., 2008; Wallwork *et al*., 2014). Genger *et al*. (2005) has reported a further major QTL *Rrs15* (also derived from *H. spontaneum*) located at 626.3 Mb on chromosome 7H. Cheong *et al*. (2006) reported a major QTL for scald resistance phenotyped under field conditions that overlaps with the *Rrs15* genome position.

## 6 INTROGRESSION OF SCALD RESISTANCE FROM WILD BARLEY RELATIVES

The wild relatives of domesticated barley, in particular *H. spontaneum* and *H. bulbosum*, are important sources of genetic diversity for scald resistance. The reliance on domesticated barley resistance genes exerts strong selection pressure for the corresponding virulence genes in the pathogen population, and thus wild relatives of cultivated barley are able to provide novel resistance and expand the genetic base of cultivated barley. QTLs for scald resistance discovered in *H. spontaneum* were also previously identified in cultivated barley at the *Rrs1* locus (Genger *et al*., 2003a, 2003b; von Korff *et al*., 2005). So far, from eight studies examining eight genotypes, five novel scald resistance loci have been identified from wild barley as follows. From *H. spontaneum*, *Rrs12* was mapped on chromosome 7H, possibly offering a potentially different allele of *Rrs2* (Abbott *et al*., 1992; Genger *et al*., 2005). *Rrs13* was identified on chromosome 6H from *H. spontaneum* (Abbott *et al*., 1995). *Rrs14* was identified on chromosome 1H from *H. spontaneum* (Garvin *et al*., 2000). Another novel locus for scald resistance *Rrs15* from *H. spontaneum* was mapped on chromosome 7H (Genger *et al*., 2005). From wild barley *H. bulbosum*, *Rrs16* was identified on chromosome 4H (Pickering *et al*., 2006). These five novel loci provide valuable resources to introgress and pyramid the different scald resistance genes from wild barley in cultivated barley. However, further experiments are required to investigate the allelic differences between the QTLs from wild and cultivated barley.

## 7 IDENTIFICATION OF CANDIDATE GENES AT MAJOR SCALD RESISTANCE LOCI

The successful cloning of genes at the *Rrs1* and *Rrs2* loci will advance the understanding of scald resistance. It will allow functional analysis of the genes themselves, which will also inform utilization of these loci in breeding programmes. So far, none of the major scald resistance genes has been cloned, although at least one attempt has been made to clone *Rrs1* (Oldach, 2012). The recent publication of the barley genome (Mascher *et al*., 2017) will facilitate new work in this area, as a range of tools are developed to interrogate and use sequence data.

Simply searching for candidate genes of the major scald resistance QTLs through BLAST searches of fine‐mapped marker intervals is a widely used approach (Hanemann *et al*., 2009; Hofmann *et al*., 2013; Coulter *et al*., 2019). The sequences of fine‐mapped markers at the *Rrs1* and *Rrs2* loci can be used to perform BLAST searches against the high‐confidence gene sequences on the IPK Barley Blast Server (http://webblast.ipk‐gatersleben.de/barley). This is likely to reveal a large number of candidate genes that will need further investigation. The challenge with searching for scald resistance loci using BLAST searches is that they may not be present in the scald‐sensitive Morex reference genome sequence (Coulter *et al*., 2019). Diagnostic markers have been developed for both fine‐mapped *Rrs1* (Rh4 type) (Looseley *et al*., 2020) and *Rrs2* (Hanemann *et al*., 2009). Overall 10 high‐confidence annotated genes were identified at the *Rrs1* (Rh4 type) locus although the actual candidate gene remains elusive (Looseley *et al*., 2020). However, the *Rrs1* (Rh4) locus was absent in the Morex genome (Mascher *et al*., 2017), which is cited as a major impediment to identifying the causal gene for this locus (Looseley *et al*., 2020). Detailed work by Marzin *et al*. (2016) investigated a family of putative pectin esterase inhibitor (PEI) genes at the *Rrs2* locus. The study was able to conclude that no single PEI gene of the three investigated was the *Rrs2* gene, and suggests the possibility that another known or unknown PEI gene or some combination of PEI genes may be *Rrs2* instead. The authors also suggested that the resistance gene at the *Rrs2* locus may be absent from the reference genome sequence that was generated from the susceptible cultivar Morex (Marzin *et al*., 2016).

BLAST approaches could evolve to other more targeted genome searching methods as sequence information becomes more comprehensive. Bioinformatics approaches without prior knowledge of the mapped locus may prove useful to identifying individual genes.

The location of genes from families with a known role in resistance and a common protein structure, for example wall‐associated kinases or nucleotide‐binding site leucine‐rich repeat (NBS‐LRR) proteins, could be targeted to identify new resistance genes in these families scattered across the genome. The majority of cloned disease resistance genes in plants encode NBS‐LRR proteins (McHale *et al*., 2006; Dangl *et al*., 2013; Kourelis and van der Hoorn, 2018). Cloned wheat stem rust resistance gene *Sr33* and wheat leaf rust resistance gene *Lr10* both encode a coiled‐coil (CC)‐NBS‐LRR protein (Loutre *et al*., 2009; Periyannan *et al*., 2013). A cereal NBS‐LRR bait library was designed to predict NBS‐LRR genes present in Triticeae species. MutRenSeq was developed for rapid gene cloning (Steuernagel *et al*., 2016). By using MutRenSeq, the stem rust resistance gene *Sr45* was cloned in wheat and the contig encodes a CC‐NBS‐LRR protein (Steuernagel *et al*., 2016).

However, whole‐exome capture and MutRenSeq can only identify annotated genes in reference genomes, and genes that are not in the reference genome may be missed. MutChromSeq was developed to reclone the barley *Eceriferum‐q* gene and clone de novo the wheat *Pm2* gene, and this method does not require construction of a physical reference sequence across a map interval or fine mapping (Sánchez‐Martín *et al*., 2016). All of these advanced technologies provide less expensive and faster approaches to clone the scald resistance genes in barley.

## 8 FUTURE IMPROVEMENT OF SCALD RESISTANCE WITH ADVANCED GENOMIC TOOLS

Historically, QTLs have been identified through linkage analysis of biparental mapping populations. The 148 QTLs for scald resistance reviewed and summarized here were all identified using biparental mapping populations. This method is useful for detecting large‐effect QTLs with rare alleles and has been an important tool for marker‐assisted breeding for scald resistance in barley (Lorenz *et al*., 2011b). However, other approaches, including genome‐wide association studies (GWAS) and genomic selection, may uncover new sources of resistance to scald.

GWAS are able to detect loci associated with target traits using diverse germplasm sets. This can introduce greater allelic diversity compared to biparental populations where only alleles segregating between the two parents can be evaluated (Zhu *et al*., 2008; Myles *et al*., 2009). Depending on the nature of linkage disequilibrium decay within the population under study, GWAS may facilitate higher mapping resolutions (Boyd *et al*., 2013; Sukumaran and Yu, 2014). However, GWAS are generally limited in power to detect very rare alleles or alleles with small effect sizes. Increasing the sample size can go some way to improving the power of association studies (Korte and Farlow, 2013).

So far, overall 13 QTLs from three GWAS analysis for scald resistance have been detected under natural field conditions at adult plant stage (Table 2 and Figure 2) (Gawenda *et al*., 2015; Looseley *et al*., 2018; Daba *et al*., 2019). GWAS analysis suggested *Rrs1* is the most significant effect QTL among European spring barley germplasm under natural field conditions in Europe (Looseley *et al*., 2018). Interestingly, this study included cultivars that are known to carry the *Rrs2* locus, but no QTL at the *Rrs2* locus was detected in the field. In contrast, another GWAS analysis detected two large‐effect QTLs for scald resistance on chromosome 7H (Daba *et al*., 2019). This GWAS analysis was carried out in Ethiopia by using barley genotypes from Ethiopia, ICARDA, and the USA. Novel QTLs for scald resistance on chromosomes 1H, 5H, and 7H were also identified (Daba *et al*., 2019). The results again indicate that the genetic architecture of scald resistance in barley is complex and the effects of resistance genes vary depending on environmental effects.

**Table 2:** Summary of the scald resistance quantitative trait loci (QTL) from genome‐wide association studies included in this review ordered by chromosome position giving logarithm of the odds (LOD) scores, percentage of phenotypic variation explained by the QTL in the mapping population (where reported), and marker information

Original QTL	Chr	Physical position (Mb)	LOD	%	Marker	Reference
Qsc‐1H	1H	463.8	4.4	12.3	S1H_463825184	Daba *et al*. (2019)
QA1	2H	16.5	3.8	–	SCRI_RS_155957	Looseley *et al*.(2018)
QA2	2H	656.6	3.8	–	SCRI_RS_138045	Looseley *et al*. (2018)
QTLG2H	2H	669.2	–	–	SCRI_RS_231015	Gawenda *et al*. (2015)
QA3	3H	446.9	9.9	–	SCRI_RS_221644	Looseley *et al*. (2018)
QA4	3H	512.2	4.2	–	SCRI_RS_227898	Looseley *et al*. (2018)
QA5	3H	570.9	3.2	–	SCRI_RS_138723	Looseley *et al*. (2018)
QA6	4H	10.1	3.3	–	SCRI_RS_197394	Looseley *et al*. (2018)
Qsc‐5H.3	5H	587.4	4.9	9.5	S5H_587435242	Daba *et al*. (2019)
QA7	6H	16.4	3.4	–	SCRI_RS_201251	Looseley *et al*. (2018)
QA8	7H	35.5	3.8	–	SCRI_RS_138457	Looseley *et al*. (2018)
Qsc‐7H.1	7H	84.0	6.7	15.6	S7H_84013770	Daba *et al*. (2019)
Qsc‐7H.3	7H	165.6	5.6	14.9	S7H_165483578	Daba *et al*. (2019)

Note

All 13 QTLs from three GWAS reports for scald resistance were detected under natural field conditions at adult plant stage.

Genomic selection uses marker measures of realized relatedness from whole‐genome marker profiles to model genomic estimated breeding values (GEBVs) (Meuwissen *et al*., 2001; de los Campos *et al*., 2013; Habier *et al*., 2013). A training population of phenotyped lines is genotyped together with lines without phenotypes and the relatedness of genotype‐only lines with phenotyped lines is used to model GEBVs (Heffner *et al*., 2011; Lorenz *et al*., 2011a). Genomic selection methods have been applied to other barley diseases, including fusarium head blight resistance (Lorenz *et al*., 2012; Sallam *et al*., 2015). Accurate phenotyping remains the challenge for complex traits such as disease resistance in the breeding programmes (Cooper *et al*., 2014; Zhang *et al*., 2017). By leveraging the information from difficult and expensive phenotyping through modelling GEBVs for lines without phenotypes, genomic selection may enable more rapid and inexpensive selection for multiple resistance loci (de los Campos *et al*., 2013; Desta and Ortiz, 2014), therefore genomic selection could enhance the rate of genetic gain of disease resistance traits (Lorenz *et al*., 2011b). Genomic selection approaches warrant investigation for their potential to improve scald resistance.

## 9 CONCLUSIONS

The deployment of cultivars resistant to scald and understanding *R. commune* pathogen populations has helped reduce pesticide applications globally. However, *R. commune* can change quickly to overcome the deployed resistance genes. Therefore, introgression and pyramiding different resistance genes into one cultivar is likely to be effective to enhance durable scald resistance. Overall 148 QTLs for scald resistance were summarized from 34 different studies in this review. All of these resistance QTLs were projected on the barley pseudomolecules Morex v. 2.0 2019. The genome sequence enables us to understand the physical positions of previously reported QTLs and compare QTL results between linkage mapping studies. Finally, we have summarized many of the qualitative and quantitative resistance QTLs that will be crucial for improvement of scald resistance in future global barley breeding efforts.

## Data Availability

Data sharing is not applicable to this article as no new data were created or analysed in this study.
